# Selecting Monoclonal Cell Lineages from Somatic Reprogramming Using Robotic-Based Spatial-Restricting Structured Flow

**DOI:** 10.34133/research.0338

**Published:** 2024-03-07

**Authors:** Xueping Chen, Ke Fan, Jun Lu, Sheng Zhang, Jianhua Dong, Jisheng Qin, Weihua Fan, Yan Wang, Yiyuan Zhang, Huo Peng, Zhizhong Zhang, Zhiyong Sun, Chunlai Yu, Yucui Xiong, Yan Song, Qingqing Ye, Shiwen Mai, Yuanhua Wang, Qizheng Wang, Fengxiang Zhang, Xiaohui Wen, Tiancheng Zhou, Li Han, Mian Long, Guangjin Pan, Julian F. Burke, Xiao Zhang

**Affiliations:** ^1^ Guangzhou Institutes of Biomedicine and Health, Chinese Academy of Sciences, Guangzhou 510530, People’s Republic of China.; ^2^ Institute of Mechanics, Chinese Academy of Sciences, Beijing 100190, People’s Republic of China.; ^3^ Institute of Electrical Engineering, Chinese Academy of Sciences, Beijing 100190, People’s Republic of China.; ^4^ CAS Key Laboratory of Regenerative Biology, Joint School of Life Sciences, Guangzhou Institutes of Biomedicine and Health, Chinese Academy of Sciences, Guangzhou 510530, People’s Republic of China; Guangzhou Medical University, Guangzhou 511436, People’s Republic of China.; ^5^ Biological Sciences, University of Southampton, University Road, Southampton SO17 1BJ, UK.; ^6^ School of Light Industry and Engineering, South China University of Technology, Guangzhou 510641, People’s Republic of China.; ^7^ University of Electronic Science and Technology of China, Chengdu 611731, People’s Republic of China.

## Abstract

Somatic cell reprogramming generates induced pluripotent stem cells (iPSCs), which serve as a crucial source of seed cells for personalized disease modeling and treatment in regenerative medicine. However, the process of reprogramming often causes substantial lineage manipulations, thereby increasing cellular heterogeneity. As a consequence, the process of harvesting monoclonal iPSCs is labor-intensive and leads to decreased reproducibility. Here, we report the first in-house developed robotic platform that uses a pin-tip-based micro-structure to manipulate radial shear flow for automated monoclonal iPSC colony selection (~1 s) in a non-invasive and label-free manner, which includes tasks for somatic cell reprogramming culturing, medium changes; time-lapse-based high-content imaging; and iPSCs monoclonal colony detection, selection, and expansion. Throughput-wise, this automated robotic system can perform approximately 24 somatic cell reprogramming tasks within 50 days in parallel via a scheduling program. Moreover, thanks to a dual flow-based iPSC selection process, the purity of iPSCs was enhanced, while simultaneously eliminating the need for single-cell subcloning. These iPSCs generated via the dual processing robotic approach demonstrated a purity 3.7 times greater than that of the conventional manual methods. In addition, the automatically produced human iPSCs exhibited typical pluripotent transcriptional profiles, differentiation potential, and karyotypes. In conclusion, this robotic method could offer a promising solution for the automated isolation or purification of lineage-specific cells derived from iPSCs, thereby accelerating the development of personalized medicines.

## Introduction

Human pluripotent stem cells (hPSCs) are self-renewing cells with the potential to differentiate into various functional cell lineages, and suitable for transplantation purposes and in vitro cell-to-tissue modeling [1–3], which is the ultimate key of regenerative medicine [4]. There are 2 main types of pluripotent stem cells: human embryonic stem cells (hESCs) and human induced pluripotent stem cells (hiPSCs). In regenerative medicine, utilizing iPSCs provides a tremendous advantage in avoiding ethical controversy and serves as a personalized and patient-specific cell source [5]. Nowadays, more clinical trials are initiated based on personalized hiPSCs than hESCs [1,6], and these cells are subsequently differentiated into the desired lineage-specific somatic cells for gene and cell therapy to perform essential functions.

The reprogramming of somatic cells into iPSCs involves the induction of transcriptional factor regulation (OCT4), DNA modifying agents (KLF4), and microRNA (miRNA 302) to stabilize the pluripotency in epigenetic alteration [7,8]. However, the conventional laboratory operations for personalized hiPSC culturing, identification, selection, and post-culturing expansion are labor-intensive and time-consuming. Although the overall frequency of somatic cells transitioning to pluripotency is typically low (<1%) [9], the kinetics and efficiency of iPSC generation have been improved through the regulation of epigenetic manipulation using mRNA or small-molecule inhibitors [10,11], achieving an average of 9.5% in certain somatic cell types [12]. However, the harvesting rate might be lower due to the uncertainty of manual operations. Technological advancements in iPSC selection have been made through the use of microfluidic devices or serial automated commercial equipment [13–15]. However, these approaches enable the harvest of iPSCs in a pooled population rather than as individual clones, which might cause the formation of chimeric colonies. Additionally, the low culturing viability of human iPSCs makes it impractical to use single-cell limiting dilution for subcloning [16,17]. Moreover, for post-harvest of iPSCs in subcloning or purification, it is even more tedious and labor-intensive to maintain the pluripotency for stable cell line expansion to meet the regulatory requirements of the cell therapy [18]. Therefore, to develop technologies for the harvesting and enrichment process of iPSCs, it is necessary to achieve (a) the determination of hiPSCs clonality with monoclonality, (b) a label-free and non-invasive approach, and (c) minimal manual intervention and maximum data traceability. Consequently, a cross-disciplinary approach is required to employ engineering techniques for the molecular characterization of cell lineage alterations in this heterogeneous population during the selection of hiPSCs in somatic cell reprogramming.

The typical sources of cells for somatic cell reprogramming include skin, blood, adipose tissue, and urine [19]. These cells can easily be obtained through skin biopsies, blood samples, or liposuction procedures. However, among these sources, only urine cells can be collected in a non-invasive manner with minimal post-collection manipulation [20], making them the most convenient choice for personalized medicine involving induced pluripotent stem cells (iPSCs). Our previous study utilized a hidden Markov model (HMM) to predict the formation of iPSC colonies during urinal cell (UC) reprogramming [21]. This prediction was based on time-lapse bright-field imaging analysis, which is a label-free and non-invasive method for assessing morphological changes in colonies. The changes in colony forming texture are echoed by pluripotent cell compacting, with ROCK-dependent survival mediated by E-cadherin, and pluripotent fate sustaining [22,23]. During somatic cell reprogramming, E-cadherin production is associated with the process, and remarkably, exogenous expression of E-cadherin can replace the requirement for OCT4 during reprogramming as the cellular polarity changes in mesenchymal–epithelial transition (MET) [24]. Another important adhesion molecule, β-catenin, has a central role in directing diverse intracellular functions and enhances the expression of pluripotency circuitry genes to promote somatic cell reprogramming. It is reported that the β-catenin/E-cadherin complex is directly regulated by OCT4 during the process of changing polarity [25]. Previous research has demonstrated the crucial role of E-cadherin in the regulation of early differentiation processes [24]. This process entails the loss of pluripotency and the E-cadherin is vital for preserving pluripotency across different stem cell compartments [22,24]. Consequently, the adhesive molecule's signature plays a substantial part in determining cell lineage. Studies have extensively used hydrodynamic shear flow assays, utilizing microfluidic devices or parallel flow chambers, to study cell adhesion on defined substrates and observe real-time cell detachment [13,26]. This flow-manipulated selection of cell adhesion strength is correlated with the specificity of the extracellular matrix (ECM) and can be used as an engineering approach for isolating certain cell types in the heterogeneous population.

The technical challenges associated with acquiring iPSCs in a monoclonal manner with high purity extend beyond biological approaches, such as factors or small molecules. Despite the challenges, developing a system that can harvest iPSCs label-free and non-invasive while maintaining their monoclonality is crucial for cell therapy. Here, we hypothesize that adhesion variation between cell–cell and cell–matrix is integral to somatic cell reprogramming, particularly during the epithelial–mesenchymal transition phase, and changing in adhesion signature can be utilized for cell-type selection through a specific spatial shear stress pattern in maintaining monoclonality. To test this hypothesis, we propose employing a robotic system with a precise mechanical design capable of manipulating shear stresses in a programmed manner. This automated system would enable identifying and selecting specific cell types in a heterogeneous lineage environment with a determined spatial resolution, thereby achieving monoclonality.

## Results

### Utilizing pluripotency-related integrin and cadherin patterns for wall shear stress selection of hiPSCs

**Fig. 1.**
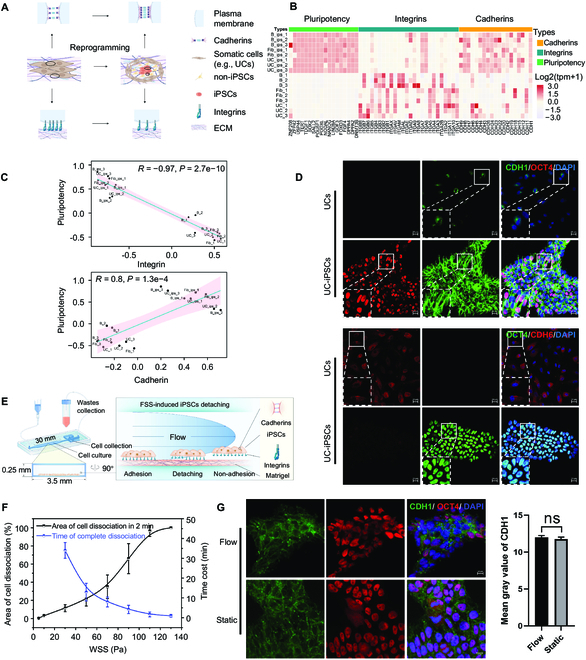
Utilizing fluid shear stress enables the selection of hiPSCs based on pluripotency-related gene expression patterns. (A) Schematic representation of changes in integrin and cadherin expression patterns during somatic cell reprogramming. (B) The heatmap analysis of ssGSEA results between somatic cells and their derived iPSCs using reference gene sets for integrins, cadherins, and pluripotency, respectively. B: blood, Fib: fibroblast, UC: urine cell. (C) Correlation analysis of pluripotency gene set with integrin gene set or cadherin gene set in somatic cells and their derived iPSCs. (D) Immunofluorescence staining of cadherins (CDH1 and CDH6) in somatic cells (UCs) and their derived iPSCs; scale bar is 20 μm. (E) Schematic of fluid shear stress-induced iPSCs colony dissociation in PPFC. (F) Correlation analysis of iPSC colony dissociation with WSS. (G) Immunofluorescence staining comparison of iPSCs cell–cell connections under static conditions or after dissociation by laminar flow. Scale bar is 10 μm.

The process of somatic cell reprogramming to generate iPSCs involves multiple changes that contribute to cell fate heterogeneity. These changes include alterations in gene expression profiles [27], epigenetic state [28,29], cell morphology [30,31], and cellular metabolism [32]. All of these factors are known to impact the MET and, thus, the characteristic transitions of adhering molecules (Fig. 1A). To investigate the generality of transitions in adhering molecule during hiPSC generation, somatic cell reprogramming was implemented using UCs, skin fibroblast cells (FibCs), and blood cells (peripheral blood mononuclear cells [PBMCs]). Next, a bulk RNA sequencing (RNA-seq) assay was carried out between these 3 types of somatic cells and their corresponding iPSCs. Figure 1B shows the single sample gene set enrichment analysis (ssGSEA) between somatic cells and their derived iPSCs using selected reference gene sets representing integrins, cadherins, and pluripotency, respectively (Fig. 1B). The heatmap illustrated that while gaining the pluripotency profile, the expression of the integrin gene panel for cell–ECM interactions decreased, in contrast with the enhanced expression of the cadherin gene panel for cell–cell interactions. This finding was further validated by examining the expressions of integrins with pluripotency genes (iPSC-related genes), which showed a negative correlation (Fig. 1C); in contrast, cadherin expression was positively correlated (Fig. 1C). Interestingly, PBMCs were reprogrammed in suspension culturing conditions to become adhering during hiPSC colony formation. Further, these bioinformatics analysis findings were confirmed using immunostaining. As expected, integrins (ITGB1, ITGA5, and ITGA6) gained higher fluorescent intensity around cell membranes in UCs and FibCs than that of the derived iPSCs (Fig. S1), whereas cell–cell interactions by cadherins were enhanced in hiPSCs (Fig. 1D and Fig. S1). Therefore, taking into account the molecular signatures identified in this study, our data support the notion that alterations in cell lineage during the process of iPSC formation may result in reduced ECM adherence of cells and increased expression of genes involved in cell–cell interactions. As such, our findings provide valuable insight for further physical characterization to manipulate these observed biological phenomena.

Subsequently, parallel-plate flow chamber (PPFC) analysis was carried out to investigate the mechanical behavior and characterization of cultured UC-derived iPSC colonies exposed under steady laminar fluid shear stress (FSS), thus allowing the examination of the colony dissociation pattern in correlation with flow changes. Therefore, the in-house developed PPFC experimental microfluidic device comprised a flow chamber for cell culturing and a separate well for collecting detached cells (Fig. 1E). The microfluidic device (Fig. S2A), which can apply a continuous steady laminar flow field (Fig. S2B), and the computational fluid dynamics (CFD) simulation results showed that the designed flow field effectively entraps cells within the collection area after dissociation (Fig. S2B and Movie S1). By manipulating various flow rates and action time, it was demonstrated that the dissociation area of iPSC colonies induced by FSS was enhanced with both increasing fluid wall shear stress (WSS) and duration. That is, the area magnitude of cell dissociation exhibited a positive correlation with WSS, whereas the time required for colony complete dissociation was negatively correlated with WSS (Fig. 1F and Fig. S2C). In addition, the time-lapse imaging analysis demonstrated that under the experimental FSS condition (5 to 130 Pa), the cell colonies exhibited a tendency to remain connected in a cell–cell format while detaching from the culture surface (Movie S2). Next, immunofluorescent staining was utilized to examine the expression pattern of cadherin subsequent to the detachment of UC-derived iPSC colonies. This detachment process was conducted by implementing FSS in the PPFC. The observed CDH1 expression pattern, which was localized at the cell–cell connecting membrane and displayed a higher immunofluorescence value, was consistent with that of the iPSCs cultivated under static conditions (Fig. 1G). This finding suggested that the cell–cell connection remained intact even after the flow selection process, which is consistent with the literature that adherence junctions in iPSCs can activate Wnt signaling through positive cadherin, thereby enhancing cell survival and proliferation [33,34]. Considering these findings, the efficacy of the proposed parabolic laminar flow for iPSCs selection and purification is highly promising.

### Specific cell lineage selection through radial shear flow generated by pin-tip-based micro-structure

During the process of cell reprogramming, the formation of iPSC colonies occurred in a randomly distributed manner within the culture dish, making it challenging to isolate specific cell types under single-clone conditions using globally oriented laminar flow-based methods, such as microfluidic devices [13] or PPFC. Despite this challenge, it was still possible to use computer vision for the identification and localization of iPSCs colonies as well as fluid mechanics for the characterization of reprogramming cells. In principle, it was feasible to generate a laminar flow field at a specific location to facilitate the selection of a single colony as required. Unlike the globally oriented laminar flow field in the microfluidic devices [13] or in the PPFC, the flow field generated by aspirating a pin-tip produces a radial flow field where the flow converged toward the center of the pin-end. Theoretically, this radial shear flow can be employed, in combination with computer vision-based localization, to isolate individual iPSC colonies that are randomly distributed within the culture dish.

**Fig. 2.**
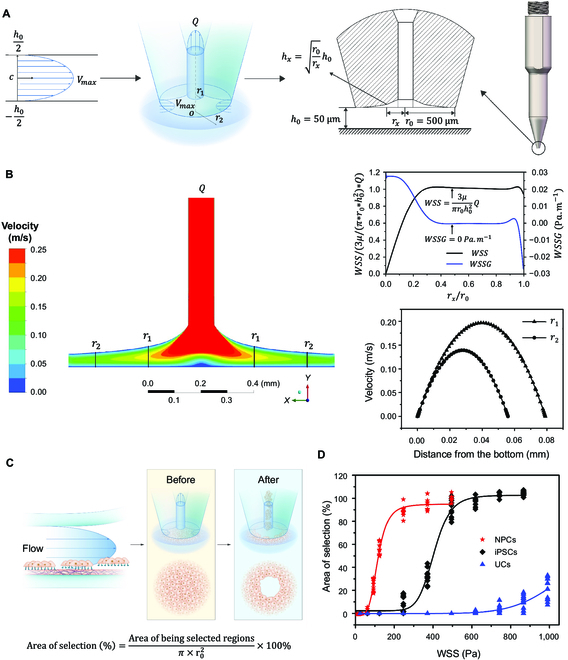
The specificity selection of adhered cells by the PTMS-FLOW. (A) Schematic of pin-tip-based micro-structure (PTMS) for generating localized radial flow and the principle of cell specificity selection. (B) The simulation of the flow field distribution based on the CFD method. The WSS is proportional to uptake flow (*Q*), whose magnitude is equal to $\frac{3\mu }{\pi r_{0}h_{0}^{2}}Q$. The micro-structure allows the pin to aspirate with a constant WSS in the radial direction above the cell culture surface. (C) The experimental principle for PTMS-FLOW-based cell colony selection using the PTMS. (D) Comparison of fluid selected area of NPCs, iPSCs, and UCs after applied PTMS-FLOW. The NPCs illustrate the lowest adhesion force, followed by iPSCs and UCs.

To apply the radial flow for single clone isolation, the present study detailed the design of a pin-tip-based micro-structure (PTMS) for iPSC colony selection, featuring a unique structural criterion aimed at generating a localized laminar shear flow as depicted in Fig. 2A. The PTMS-generated radial flow (PTMS-FLOW) was a distinctive structured flow that allows for precise generation of uniform shear stress patterns on specific regions of the culturing dish surface with the help of our robotic system. The hydrodynamic principle and derivation method for this design were elaborated in Materials and Methods. The fluid continuity equation illustrated that with the distance from the PTMS to the cell culture surface *h*_0_, and given that the height of the curve contour of the PTMS along the radial direction vertical from culture surface satisfies $h_{x}=\sqrt{\frac{r_{0}}{r_{x}}}h_{0}$, this PTMS enabled the pin to aspirate with a constant WSS in the radial direction above the cell culture surface. Thus, this WSS was proportional to uptake flow rate (*Q*), and the magnitude was equal to $\frac{3\mu }{\pi r_{0}h_{0}^{2}}Q$. To verify the rationality of this structure, we took *h*_0_ = 50 μm and *r*_0_ = 500 μm, then simulated the flow field distribution using the CFD method (Fig. 2B). The results illustrated that the PTMS-FLOW exhibited a feature with a stable radial flow field, and the WSS of the fluid along the radial direction remained constant; in addition, the area of the constant WSS generated by the PTMS achieves more than 90% coverage ($\pi r_{0}^{2}$). Moreover, the value of the WSS gradient in most of the area along the radial direction was 0 Pa ∙ m^−1^. The mechanical movement or unevenness of the cell culture surface might cause variation and allow the PTMS to shift away from the optimal distance and caused an unpredictable flow field, as *h*_0_ might become a variable. This scenario was assessed to investigate the WSS value as *h*_0_ = 50 μm, based on the CFD simulation, with variations of ±5 μm and ±10 μm (Fig. S3). As agreed with the equation, while the offset values are +5 μm, +10 μm, −5 μm, and −10 μm, the corresponding maximum changes of the WSS value were −2.46%, −4.38%, 2.46%, and 5.64%, respectively, with the expected correlation (Fig. S3F). Hence, once *h*_0_ was restricted to within ±5 μm, the absolute value of the maximum horizontal variation of the WSS can be maintained within 3% as expected, thus maintaining the stability of the flow field in the radial direction for functional applications.

Subsequently, to validate the effectiveness of PTMS-FLOW in selecting cell lineages, the above-mentioned micro-structure was utilized to isolate various types of cell colonies, including neuron progenitor cells (NPCs), hiPSCs, and UCs. The experimental approach for cell colony selection was facilitated by the micro-structure with an outer contour of *r*_0_ = 500 μm, situated at the tip of the PTMS (Fig. 2C). The evaluation criterion for selection efficacy was based on the tenets of a biomechanical principle. Specifically, the selected cell area exhibited a negative correlation with the cell adhesion strength under a controlled period (1 s) of various WSS forces (0 to 1,000 Pa). After 5 days of culturing, monolayer NPC, iPSC, and UC populations (targeted area >500 μm) were operated using the PTMS-FLOW generated by the PTMS, and the selected area versus FSS was plotted (Fig. 2D). As the results showed, NPCs demonstrated the lowest adhesion force, and the PTMS-FLOW-mediated selection threshold was initiated as 124 Pa, followed by that of iPSCs at approximately 248 Pa; UCs demonstrated the highest adhesion force, at 744 Pa (Fig. S4). This finding illustrated that the PTMS-FLOW generated at the aspiration pin-end via the PTMS could sufficiently distinguish different cell lineages, and could potentially select iPSCs clones in demand.

### Robotic system for automated monoclonal hiPSC generation in somatic cell reprogramming

**Fig. 3.**
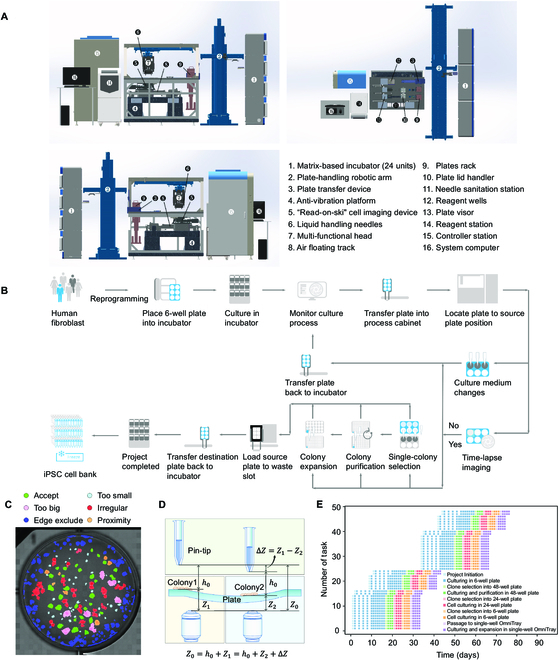
Robotic system for selecting single iPSCs colony. (A) Mechanical structure and layout of the robotic system for single iPSC colony selection and culturing, shown from different orientations (front, back, and top). The key functional modules of this system include 6 compartments, matrix-based incubator, the plate-handling robotic arm, the cell process cabinet, the reagent station, the controller station, and the system computer, which are labeled with numbers. The precisions of the *X*, *Y*, and *Z* axis of the multi-functional head (the linear motor) are ±0.6 μm/250 mm, ±2.0 μm/250 mm, and ±2.2 μm/50 mm, respectively. (B) The workflow of the iPSC colony selection and culture system. (C) The results of conducting segmentation on a detection map; each resulting segment represents a detected colony. Different colors indicate different maturation periods, which are listed at the top of the map. (D) The principle of an adaptive feedback controlling mechanism is used to ensure the aurate *Z*-axis distance of the PTMS. (E) Task scheduling. The entire process was managed by programmed scheduling for parallel operation.

In order to implement the automated capacity of the single-colony iPSC selection for monoclonality, a robotic cluster was designed and assembled in-house to accomplish the process in a semi-unmanned manner. The system consisted of 6 functional compartments, including the 24 personalized matrix-based cell culturing incubators (①), the transfer linear robotic arm with a plate-handling grip (②), the cell process cabinet (③ to ⑬), the reagent station (⑭), the controller station (⑮), and the system computers (⑯) (Fig. 3A). The entire serial robotic system was regulated via a lower embedded control board using ARM + DSP + FPGA multi-core integration for high-speed control (see Materials and Methods). In brief, the ARM handled communication with the master system and scheduling, the DSP core performed multi-axis synchronized motion control trajectory planning and algorithm processing between functional modules, and the FPGA synchronized 23-axis interpolation within the 0.5-ms main loop [35]. Hence, the entire somatic cell reprogramming and post-iPSC expansion-related cell culturing process, including time-lapse-based high-content microscopic imaging, media changes, synchronized colony formation detection, and single iPSC colony selection and expansion, were all scheduled through the modulated program for system controlling and computer vision (Fig. 3B and Fig. S5).

Within 24 h after the UCs were transfected using the non-integration vector system to initiate the somatic cell reprogramming, as reported previously [21,36], the 6-well plate was placed in one of the matrix-based incubators. Time-lapse imaging was performed with media changes for the entire culturing well, followed by computer vision for colony detection. As we reported previously, we combined the non-invasive and label-free iPSCs colony detection and maturation prediction procedure [21] with the trans-illumination-based “read-on-ski” imaging technology [37] for improved imaging quality, speed, and accuracy (Fig. S6A and Movie S3). To ensure iPSC monoclonality, the computer vision with machine learning system detected the iPSC formation and maturation periods. This digitized colony formation process calculated the proximity between colonies to eliminate the merged colonies or colonies with low proximity that might interfere with the PTMS-FLOW during the selection process (Fig. S6B and C). Next, qualified iPSCs colonies with located *x* and *y* coordinates were generated to guide the linear robotic arm to operate the cherry-picking selection (Fig. 3C). Moreover, to overcome variation in the culture plate or colony thickness, an optical autofocus device was implemented to calculate ∆*Z*. This adaptive feedback controlling mechanism was implemented to ensure the accurate *z* distance (*h*_0_ = 50 μm) for iPSC selection via PTMS-FLOW (Fig. 3D) in a robust manner. Finally, the entire process was managed by a pre-programmed scheduling algorithm for parallel operation, as the entire reprogramming process was divided into 9 automation tasks (Fig. 3E). Parallel projects could be initiated, 14 reprogramming missions could be started simultaneously, and the variation of colony maturation ranged within 2 to 3 days. After the monoclonal iPSCs colony selection, the system performed the iPSC culturing and purification procedure in multi-well plates (see Materials and Methods). Afterward, selected clones were further expanded (to 24-well plate and 6-well plate) and single-well OmniTray. Twenty-four reprogramming projects can be processed within 50 days when using the full capacity of the system.

### PTMS-FLOW-mediated cell lineages selection for refined hiPSCs generation

**Fig. 4.**
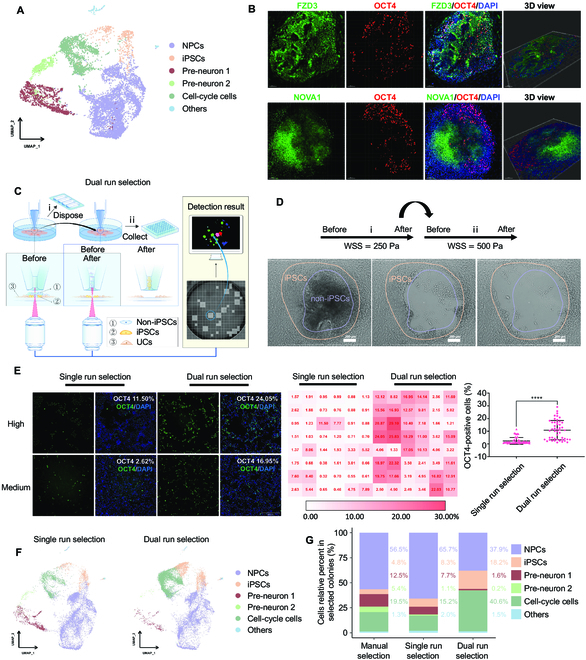
PTMS-FLOW technology enhances iPSC generation by cell fate selection. (A) UMAP embedding showing cell types of the selected colonies through manual selection. NPCs: neuron progenitor cells, iPSCs: induced pluripotent stem cells. (B) Immunofluorescence staining of reprogrammed colonies on Day 15. Top: FZD3 (green), OCT4 (red), and DAPI (blue); bottom: NOVA1 (green), OCT4 (red), and DAPI (blue). The confocal 3-dimensional (3D) datasets were processed with IMARIS software for 3D view; scale bar is 100 μm. (C) Schematic representation of the dual selection mode to obtain colonies. (D) Bright-field images of selected colonies using the dual selection mode. Scale bar is 200 μm. (E) OCT4 staining of selected colonies (left: single selection mode; right: dual selection mode; the OCT4 positive is represented iPSCs); High and Medium represented the fluorescence value of OCT4 staining at the high level and middle level in the corresponding selection mode, respectively. The data on the right side is the quantitative analysis of the fluorescent value of the OCT4 staining. ****P < 0.0001; scale bar is 200 μm. (F) UMAP plot analysis of the cell types from single run selection and dual run selection. (G) Comparison of the proportion of cell types (NPCs, iPSCs, Pre-neuron 1, Pre-neuron 2, Cell-cycle cells, and others) among manual selection mode, single run selection mode, and dual run selection mode.

As demonstrated, the robotic system we developed can provide an unmanned approach to avoid manual operation errors and increase the throughput of parallel somatic reprogram projects. However, even though this automated robot can perform precise iPSC identification and achieve PTMS-FLOW-mediated cherry picking of monoclonal iPSCs colonies in a way not previously reported, it cannot eliminate the heterogeneity of cell lineages during the reprogramming process for the purpose of enhancing the purity of harvested iPSCs. On the other hand, as demonstrated above, the PTMS-FLOW can distinguish between NPCs, hiPSCs, and UCs. Interestingly, based on the single-cell RNA-Seq (scRNA-Seq) data of a manually selected iPSCs colony [36], the heterogeneity of the cell types was identified (Fig. 4A and Figs. S8A and F) as NPCs (56.5%), cell-cycle cells (19.5%), and pre-neuron 1 (12.5%) and 2 (5.4%), with iPSCs accounting for only 4.8%. This result was in line with previous studies, which suggested that the cell population of iPSCs constitutes a minority within somatic cell reprogramming colonies, and the process of somatic cell reprogramming can result in generating numerous NPCs and cell-cycle cells [12,38]. Through further analysis, it was discovered that the CDH1 gene shared expression clusters with POU5F1 (found in iPSCs) in the UMAP, while the CDH6 gene was found in an expression cluster with NPC characteristic genes FZD3 and NOVA1 (Figs. S7 and S8). This scRNA-Seq data confirmed that pluripotent gene expression was correlated with the adhesion molecules, which can be used to characterize the cell fate of iPSCs and NPCs.

Understanding these cell lineage markers in correlation with the adhesion molecules rationalized the efficacy of PTMS-FLOW for distinctive cell lineage selection. Moreover, the texture analysis of colony formation during the cell reprogramming process via the computer vision system prompted NPCs and NPC-like populations to be overlaid on the iPSCs. This observation was confirmed using immunostaining and confocal imaging with z-stack scanning, which showed that in the computer vision-detected colony, OCT4+ cells were mainly distributed at the bottom layer and peripheral area as a monolayer. In contrast, FZD3+ and NOVA1+ cells were mainly distributed at the top of the colony in a multilayer stack (Fig. 4B). This finding echoed the OCT4+ population as the minority population with a layer-based structural distribution in the somatic cell reprogramming colonies, which was distinctive from the NPCs or NPCs-like majority. Moreover, NPCs were less adherent in the culture dish than that of the iPSCs at the bottom layer; this phenomenon can be applied to the separation of cell lineage based on fluid shear force. Consequently, using molecular characterization and the robotic process, a dual selection strategy was developed (Fig. 4C). During the dual mode, guided by computer vision, the first run of PTMS-FLOW (iWSS = 250 Pa) was applied to eliminate the less adhesive multi-stacked cells on the top, after which an image was taken, followed by the second run of selection (iiWSS = 500 Pa) (Fig. 4D). The bright-field imaging of the colony pattern obtained prior to run PTMS-FLOW was similar to FZD3 and NOVA1 staining; as shown above, the OCT4 iPSCs staining pattern was consistent with the cell distribution after the first run of PTMS-FLOW (WSS = 250 Pa).

To further validate the dual selection strategy in the batch mode, a comparative investigation was carried out. In brief, after the computer vision detection in the 6-well plate, 96 out of 200 qualified somatic reprogrammed colonies were selected using the conventional single run (48 clones/WSS = 500 Pa) (Fig. S9A and B) or dual run (48 clones/iWSS = 250 Pa/iiWSS = 500 Pa) selection mode into one 96-well plate. Subsequently, high-content imaging was performed in the 96-well plate for OCT4 staining on day 7 of post-selection culturing (Fig. S9D). The OCT4 staining results illustrated that the proportion of OCT4+ cells was significantly higher in the dual run selection mode (ranging from 1.21% to 29.10%, with an average of 10.84%) compared to the conventional single run selection mode (ranging from 0.32% to 11.50%, with an average of 2.38%) (Fig. 4E). In fact, the highest proportion of OCT4+ cells in the dual run selection mode showed 2.5 times higher than that in the single run selection mode, and the average proportion was 4.6 times higher (Fig. 4E). In addition, this finding was further verified via scRNA-Seq data indicating that conventional single run selection mode (WSS = 500 Pa) yielded an iPSC population ratio 2.2 times smaller (*P* < 0.0001) (8.3%) than that obtained using dual run selection mode (iWSS = 250 Pa/iiWSS = 500 Pa) selection of iPSCs (18.2%) (Fig. 4G). Furthermore, the use of the robotic platform led to achieving a significantly higher iPSC population ratio compared to the manual selection (4.8%). In addition, the iPSCs generated through the robotic-based dual run selection mode demonstrated a purity 3.7 times greater than the manual method (Fig. 4G). On the other hand, the NPCs and the pre-neuron population was reduced to less than 40% in dual run mode (iWSS = 250 Pa/iiWSS = 500 Pa), compared to 74.5% in the conventional single run mode of selection (WSS = 500 Pa) and 74.4% in the manual selection. This agreed with the scRNA-Seq data in the first selection run (iWSS = 250), which indicated that approximately 60% of the NPC and pre-neuron population were eliminated (Fig. 4F and G and Table S1). Moreover, as demonstrated in Fig. 2D, NPCs were more sensitive to the WSS; hence, in the first run’s selected samples, the NPCs and the pre-neuron population were significantly larger compared to the proportion in dual selected instances (*P* < 0.0001) (Fig. S9C and Table S1). Thus, this negative selection for potentiation of iPSCs purity was also applied to eradicate cells that lost pluripotency (differentiated non-iPSCs) during the iPSC expansion process (Fig. S10A). Interestingly, scRNA-Seq data from the negatively selected cell population (WSS = 250 Pa) showed lowered integrin with cadherin expression and pluripotent genes (Fig. S10B), which negatively correlated with the yield of iPSCs (Fig. S10C). Finally, after automation-facilitated dual selection, purification, and expansion, iPSCs were characterized through OCT4, TRA-1-60, and SSEA4 staining, followed by the karyotyping assay. The acquired findings showed normal immunofluorescence staining patterns as well as intact chromosomal profiles (Fig. S10D and E). After identification of the karyotypes, exogenous transgenes and integration elements were also not identified (data not shown). Lastly, iPSCs were injected into severe combined immunodeficiency mice to confirm the presence of cell types derived from all 3 embryonic germ layers, and the pluripotency was confirmed (Fig. S10F).

## Discussion

**Fig. 5.**
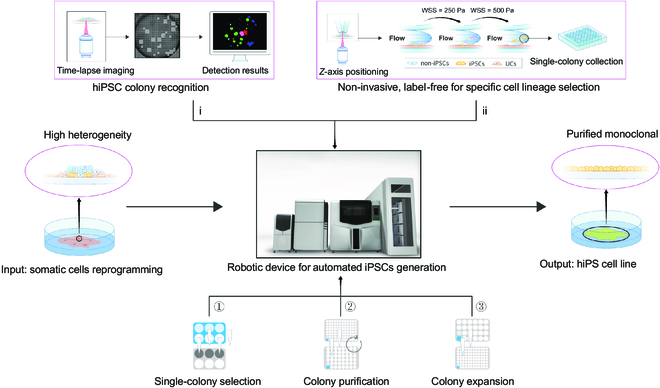
Overview of the robotic system for the iPSC colonies selection.

In this study, we developed a robotic system with a novel micro-structure that is capable of generating PTMS-FLOW, which enables distinctive cell lineage selection that can process iPSCs in a single colony. This advanced technology can be implemented as the functional payload in an automated system, capable of seamlessly integrating somatic cell reprogramming, characterization, and purifying the resulting cells (Fig. 5). This first in-house developed robotic platform offers several benefits. Firstly, it automates iPSC generation (post transfection) and allows monoclonal cell lineage selection in a label-free, enzyme-free, non-invasive, and population-based manner. Moreover, this platform also provides several systematic advantages over other technologies and developments in commercial settings, as well as the μSHEAR microfluidic chip in literature [13,18,39–41]. These advantages include high throughput, selection with monoclonality as an individual clone, imaging assurance of monoclonality, multiple selections using different FSS at the same clone, and more (Table S2).

In comparison to other research efforts, our study offers a comprehensive solution for the entire iPSC generation process following induction of reprogramming. We placed a special emphasis on our technical approaches, which adhere to regulatory requirements (non-invasive/label-free/traceable) for downstream clinical applications. Achieving homogeneous yield of seed cell under single colony conditions is a crucial criterion for ensuring the safety of cell therapy in therapeutic applications [42]. As we know, iPSCs serve as an ideal seed cell source for personalized and patient-specific solutions in pathogenesis studies (disease modeling) [2,28], drug screening (organoids) [43], and transplantation therapies (chimeric antigen receptor cell therapies, e.g., induced T cells/induced natural killer cells, induced mesenchymal stromal cells) [44–47]. Moreover, using iPSCs can help circumvent ethical debates that may arise. Our gene expression profile analysis, including single-cell data, agreed with the literature’s finding that the iPSC population is a minority (<5%) during the reprogramming process, whereas our scRNA-Seq analysis showed that the NPCs and pre-neuron population were greater than 70%. Interestingly, we found that iPSCs exhibited increased cadherin expression in comparison to other cell lineages and the iPSCs even maintained a connected multicellular patch under the WSS-induced dissociation experiment (Fig. 1D, Fig. S1, and Movie S2). This is consistent with the previous work that cadherin is the most prominent molecular signature that distinguishes iPSCs from cells with diminished pluripotency cells (such as NPCs or randomly differentiated populations during the iPSCs expansion) where OCT4 expression correlates with cadherin expression [24]. In this study, the computer vision-guided localized PTMS-FLOW can be generated using an air displacement pump (closed-loop controlled) in conjunction with the PTMS to create a wide range of precise radial shear flow magnitudes (1 to 1,000 Pa). As we know, eliminating the heterogeneity of cell lineages during the somatic cell reprogramming process is a challenging task [9,12]. Hence, it is not feasible to obtain pure iPSCs from the original reprogramming colonies. However, cell heterogeneity is associated with variations in cell adhesion strength, making it possible to distinguish between different cell types using shear flow [13,18]. Therefore, in this study, cell lineages can be selected by adjusting the magnitude of PTMS-FLOW based on different adhesion strengths of cells. Significantly, this capability enables the sequential selection of different cell lineages by PTMS-FLOW, thereby maximizing the iPSC purity per colony. Thus, the robotic-based PTMS-FLOW for the selection of iPSCs from somatic cell reprogramming colonies holds great significance.

Reprogramming and cell lineage determination resulting from epigenetic regulation contributes to functional variance among iPSCs [10]. Therefore, pool selection using polyclonal or manually derived iPSCs lines, owing to technical issues that may obfuscate intrinsic genotypic variation, might only observe the differentiation bias until a particular lineage is reached. The limiting dilution of 0.7 cells per well is the conventional approach for obtaining a single cell per well for subcloning, which may be inevitable or compulsory. Controversially, in hiPSCs, disruption of cadherin-related adhesion junction dynamics between hiPSCs interaction can affect beta-catenin Rho-associated kinase activation [33,48], which can ultimately impact multiple signaling pathways involved in proliferation and differentiation. Limiting the dilution of disassociated human stem cells can cause premature termination of cell proliferation or lost pluripotency, which may lead to epigenetic variation. PTMS-FLOW-based subcloning and purification offer an advantage by maintaining cell–cell interaction in hiPSCs, such as CDH1, while eliminating the population with unstable pluripotency during entire expansion stages to produce monoclonal-selected iPSCs with reduced epigenetic variation [49].

In the current study, we did not achieve a yield of iPSCs over 30% during the initial monoclonal isolation. However, the use of PTMS-FLOW during the purification process maintained a highly homogeneous environment for these cells. The same concept might be more beneficial for iPSC differentiation into other cell types for functional cells in relation to lineage specificity and ECM receptor or adhesion molecular signatures with the specific phenotypical cellular features, such as neural rosettes, hemogenic endothelial cells, and hepatic progenitor cells. Moreover, population-based subcloning could be a preferable solution for personalized gene editing of iPSCs, which serves as a platform for gene and cell therapy.

In summary, we present a robotic platform technology for the automated generation of monoclonal hiPSCs in a non-invasive, enzyme free, and label-free manner, which exhibited a high specificity for cell lineages selection based on PTMS-FLOW. This technology is not limited to automatically potentiating hiPSC harvest purity; it also holds great potential in hiPSC differentiation for functional cell generation with broad application in regenerative medicine.

## Materials and Methods

### System mechanical design

This in-house robotic system was specially engineered with 6 compartments, including personalized 24 matrix-based cell culturing incubators, the transfer linear robotic arm with plate handling grip, the cell process cabinet, the reagent station, the controller station, and the multiple system computers (Fig. 3A). Each incubator contained 3 hold plates, which can manually insert/obtain the multi-well plate from the front; the plate handling system shall be operated by the transfer arm with the grip. The robotic arm and cell process cabinet were operated via a linear motor (TL09/12N + magnet plate), which was part of a constructed series robotic arm with an optical encoder (RELAIN30U1A). This allowed for precise and accurate movement of the multifunction head. The controlling framework for the cell process cabinet was operated by ARM + DSP + FPGA integrated multi-core integrated for high-speed feedback. The positioning precisions of the *X*, *Y*, and *Z* axis of the linear motor are ±0.6 μm/250 mm, ±2.0 μm/250 mm, and ±2.2 μm/50 mm, respectively. The software core algorithm utilized by the control board can achieve 23-axis synchronous interpolation control and various complex computations within the 0.5-ms main loop, the same controlling board mentioned previously [35]. In addition, this controlling board managed plate handling, shaking, liquid handling, temperature control, plate rack, and the high-content imaging device, as we reported previously [21,35,37]. In particular, the liquid handling for media changes, and the PTMS-FLOW was generated by the air displacement pump (TECAN Pump, Cavro®) with an encoder motor (volume: 2.5 to 1,000 μl, speed: 2.5 to 1,000 μl/s). The reagent station was facilitated by the 4 Hamilton pumps (Hamilton, PSD/6 Precision Syringes Pump) to supply PBS, 70% ethanol (shared one pump), 2 types of cell culture media, and Matrigel. The iPSC detection and other cell-type classification utilized a computer vision system with enhanced imaging processability powered by Dual Intel(R) Xeon(R) CPU E5-2696 v3 @ 2.30GHz with 64 G memory and GeForce GTX TITAN X. The robotic mechanical movement was supported by a computer: Dell T3640 Tower Workstation under a Windows 10 operating system, including i7-10700 (8-Core/16-Thread CPUs) with 64 G memory, 512 GB SSD hard disk, and 4 TB HDD hard disk. An in-house developed Python code was used for image analysis and for generating the location information of colonies, which was used for iPSC colony detection.

### Automated multi-well plate coating

The multi-well plate was coated with a Matrigel solution (BioCoat, #354277) that had been diluted at a 1:200 ratio and mixed with pre-chilled DMEM/F12 (Cytiva, #SH30023.01) as the coating solution. This was carried out in the temperature-controlled media station, and once the plate had been coated, it was immediately placed in the process cabinet, where it was kept at 4 ± 0.3 °C on a cooling module. Multi-well plates (Greiner 96, 48, 24, 6, or Nunc Omnitray) were coated with varying volumes of coating solution per well (50, 200, 450, 1,000, and 6,500 μl) and incubated in the process cabinet at 36.2 ± 0.6 °C for 1 h using a liquid handling and intermittent shaking module (every 10 min).

### Automated iPSC clone selection based on PTMS-FLOW

Clone selection from a source 6-well plate to a destination plate (multi-well plate of a 48-well plate or a 96-well plate) was performed with a pre-programmed workflow. To initiate this process, the loader for the destination plate (specifically a 6-well plate) scanned for the presence of a multi-well plate and verified the bar code for a pre-coated registered plate. Once the source and destination plates were identified, the 6-well plate containing the reprogrammed clones was placed into the designated slot for further operation. Then, the remaining Matrigel solution in the coated multi-plate (48-well or 96-well) was replaced with mTeSR1 (200 μl or 100 μl, STEMCELL Technologies) per well. After the entire source plate imaging and colony recognition, the PTMS-FLOW strategy was implemented, and qualified clones in the 6-well plate were selected automatically according to the robotic program. Destination 48-well plates were returned to the automated incubator and the source plate was moved to the waste plate loader, which was managed daily.

In this study, a dual run selection strategy was developed. During the dual run mode process, firstly guided by computer vision, the first run of the Tech Pump was applied to eliminate the lower adhesion multi-stacked cells on the top by using lower shear stress, after which an image was taken, followed by the second run of selection to obtain higher adhesion cells by using higher shear stress (Fig. 4C).

### Automated negative selection for iPSC purification based on PTMS-FLOW

The post-selected iPSCs were purified using a process similar to the clone selection procedure. The multi-well plate (either 48-well or 24-well) was loaded into the imaging stage, where imaging and recognition of iPSC colonies as well as non-iPSC detection were carried out. After this, the PTMS-FLOW strategy was implemented to negatively select the non-iPSCs. Due to the limited space, the edged located cells were processed with the PTMS-FLOW with the closest nozzle positioning. The PTMS-FLOW was applied 3 times repetitively at each negative selection position, as coded in the software. The media replacement was carried out with mTeSR1 (200 μl or 500 μl) per well of a multi-well plate (48-well or 24-well), before being placed back into the matrix incubator via the robotic transfer arm.

### Automated iPSCs passaging and expansion

An automated system was utilized to conduct a series of liquid changes for passaging iPSCs for expansion. The system had a pre-programmed working flow that was coded with the same logic script segment for robotic movement from a 48-well plate to a 24-well plate or from a 24-well plate to a 6-well plate or from a 6-well plate to an Omnitray with different ADP pump arrangements. In summary, the rouse plate was washed twice with PBS (VivaCell, #C3580-0500) with liquid handing before the addition of a Gentle Cell Dissociation Reagent (STEMCELL Technologies, 100-0485) (48, 24, and 6 multi-well plate—200, 450, and 1,000 μl) for 6 min in the cabinet without the involvement of shaking. While the incubation, pre-coated destination plate was dispensed of mTeSR1 (24 and 6 multi-well plate or Nunc Omnitray—200, 2,000, and 11,000 μl per well) per well. After the incubation, the Gentle Cell Dissociation Reagent in the source plate was removed, and replaced with the same volume mTeSR1 per well. Then, the ADP pump was cycled for dispensing and aspiration 3 times to disassociate the cells, which intermitted with the plate movement. Following that, liquid handling transferred all the cell suspension solution into the destination plate. After the passaging procedure, the destination plates were returned to the automated incubator.

### Cell culture and reprogramming

Human UC culture and reprogramming were performed as our previously published protocol [21,36]. Briefly, the procedure involved co-transfecting 2 pCEP4 episomal plasmid containing the OCT4, SOX2, KLF4, and the miR302–367 via nucleofection (Amaxa Basic Nucleofector Kit with the T-020 program, Lonza). After 8 to 10 days post-transfection, the culture medium was supplemented with 4 inhibitors (the inhibitors were 0.5 μM A8301, 0.5 μM PD032590, 3 μM CHIR99021, and 0.5 μM thiazovivin), following which the medium was changed to mTesR1.

### Single-cell RNA sequencing

Single-cell RNA sequencing (scRNA-seq) was performed using 10X Chromium single-cell platform (10X Genomics). Preparation of single-cell suspension and sequencing library was performed as our previous study [36].

Single-cell sequencing and bioinformatic analysis were performed according to a protocol described previously and summarized briefly below [36]. The raw reads obtained from scRNA-seq were aligned against the GRCh38 reference genome using Cell Ranger (version 6.1.1, 10X Genomics). The downstream analysis (which include normalization, dimensionality reduction, and clustering MapQuery) for each sample was conducted mainly by the Seurat package (version 4.1.1) after cell filtering.

To demonstrate the statistical significance of the cell proportion differences between each clone selecting method, we used the phyper function from the stats R package (version 4.0.3), as suggested in previous work by Sankowski et al. [50]. Furthermore, we used the Benjamini–Hochberg method to correct multiple testing burdens.

### Bulk RNA-seq analysis

#### Bulk RNA-seq analysis for Fig. 1

Total RNA was extracted from cell by using TRIzol reagent (Invitrogen, 15596018). The bulk cDNA sequencing library construction, sequencing, and bioinformatics analyses were conducted as previously described in our published work [36].

#### Bulk RNA-seq analysis for Fig. S10

For bulk RNA sequencing in a small number of cells (50 to 500), we collected the cells using a robot, briefly spun them down at 500×*g*, and then performed reverse transcription and pre-amplification using the Smart-seq2 protocol [51]. All primers (Genewiz) used for reverse transcription and pre-amplification were biotinylated. We performed post-PCR cleaning with Ampure XP Beads (Beckman Coulter, A63881) at a ratio of 0.8/1 (beads/cDNA). To quantify the cDNA, we used PicoGreen (Yeasen, 12641) with FlexStation3 (Molecular Devices) and assessed the quality busing Qsep100 with S2-Standard Cartridge Kit (BiOptic). Following this, sequencing libraries were generated using the TruePrep Flexible DNA Library Prep Kit for Illumina (Vazyme, TD504) and sequenced them using the Illumina NovaSeq 6000 sequencer.

### Immunofluorescence staining analysis

UCs-electroporated were seeded in chambered microscope slides and formed clones after 15 days. The selected cells from both single-run and dual-run experiments conducted using the robotic system were collected in 96-well plates. UCs, Fibroblasts, UCs-iPSCs, and Fibroblast-iPSCs were seeded in separate chambered microscope slides. All the samples were fixed with 4%PFA for 15 min at room temperature, permeabilized with 0.2% Triton X-100 in PBS for 15 min, and blocked with 10% fetal bovine serum (FBS) for 30 min. The cells were then incubated with primary antibodies overnight at 4 °C. After rinsing with PBS, the cells were stained with secondary antibodies at 1:1,000 for 1 h at room temperature in the dark, followed by 4′,6-diamidino-2-phenylindole (DAPI) staining. For iPSC clones derived from UCs, the primary antibodies OCT4, FZD3, and NOVA1 were used to distinguish iPSCs and neural stem cells/pre-neurons. Multiple images were captured using LSM 800 (Zeiss) and 3D simulation was performed with Imaris (Oxford Instruments). To obtain a ratio of iPSCs and for image and data analysis, the primary antibody OCT4 was used as a marker of iPSCs in 96-well plates using the ImageXpress Micro Confocal (Molecular Devices). The primary antibodies ITGA5, ITGA6, ITGB1, CDH1, CDH6, and OCT4 were used in chambered microscope slides seeded with UCs, Fibroblasts, UCs-iPSCs, and Fibroblast-iPSCs. The images were taken by LSM 800 (Zeiss). Additionally, the primary antibodies OCT4 and CDH1 were used for FSS-induced iPSC colony dissociation and the images were taken by LSM 710 NLO (Zeiss). The list of the antibodies used in the paper is shown in Table S3.

### The PPFC design and fabrication

The PPFC (a microfluidic device) was used for studying the iPSC colony dissociation pattern in correlation with FSS changes. The device (length × width × height = 30 mm × 3.5 mm × 0.25 mm) was firstly designed based on the CFD method and then fabricated through the precision machining technique. The WSS in this PPFC is defined by the formula WSS = 6*Qμ*/(*h*^2^*w*) [52], where *w* is the width of the channel, *h* is height between plates, *μ* is the fluid dynamic viscosity and *Q* is the fluid volume flow rate. The polydimethylsiloxane (PDMS) prepolymer was mixed with its cross-linker at a 10:1 ratio and poured onto the fabricated model [53], which was then allowed to cure in a 90 °C oven for 1 h. A syringe was used to punch inlet and outlet holes for the fluidic channel of the PPFC device. In this study, a glass slide was applied to bond with the PDMS copy to obtain the PPFC channel. After oxygen plasma treatment of both the glass slide and the PDMS copy, they were brought into contact, and placed in an oven at 90 °C for 3 h to achieve permanent bonding.

### FSS-induced iPSCs colony dissociation experiments in PPFC

To disinfect the cell culture channel of the PPFC chip, it was treated with 75% alcohol for 10 min. The channel was then rinsed at least 5 times with PBS to ensure no alcohol remained. Next, 120,000 iPSCs (100,000/cm^2^) were suspended in mTesR1 medium and plated on the Matrigel (Corning, 354277)-coated PPFC chip. After the cells were cultured for 6 days, the adhered cells were subjected to an FSS-induced iPSC colony dissociation experiment using the following steps. (The medium was changed twice daily during this period.)

The schematic of this study is shown in Fig. 1E. The adhered iPSCs in the PPFC were dissociated using fluid flow with varying shear stress levels. In this study, the magnitude of the FSS in PPFC was carried out by the formula WSS = 6*Qμ*/(*h*^2^*w*) [49], where, *Q* is the fluid volume flow rate controlled by syringe pump. The experiment consists of 3 main steps for the process: (a) placing the PPFC device on the real-time monitoring microscope with a 10× lens; (b) adjusting the pump parameters based on the shear stress formulation for setting different flow rate *Q*; and, lastly, (c) the dissociated cells were then collected in the cell collection well and subsequently were transferred to ibidi chamber slides for further immunofluorescence staining analysis. The method of immunofluorescence staining was the same as demonstrated above. In theory, when the FSSs generated by different instruments are equal, the cells responding to the shear stress should respond similarly. Therefore, the experimental findings in the PPFC device can serve as a valuable guide for the design of custom structures in the next research.

### The hydrodynamic principle for the PTMS design

The objective of this section is to achieve a parabolic flow field analogous to that observed in the PPFC, which is utilized for selective harvesting of the localized-based monoclonal iPSCs. Therefore, this part of section focuses on engineering a localized micro-structure to create such a flow. According to the hydrodynamic principle, the flow pattern between 2 parallel plates is a parabolic profile under the laminar flow condition, as shown in the left panel of Fig. 2A. Moreover, the velocity profile between 2 parallel plates can be expressed as: $V=-\frac{4V_{\max}}{h_{0}^{2}}h^{2}+V_{\max}$, where *V_max_* is the maximum velocity in the middle of the channel and *h*_0_ is the height of the channel. Therefore, we can have $\frac{\mathrm{dV}}{\mathrm{dh}}=-8h\frac{V_{\max}}{h_{0}^{2}}$, and the WSS in the surface of the flow channel can be expressed as: $\mathrm{WSS}=4\mu \frac{V_{\max}}{h_{0}}$, where *μ* is the fluid dynamic viscosity.

In this study, the PTMS that can be used to generate a localized flow was designed for single iPSC colony selection based on the principle of hydrodynamics. The structural design principle of the PTMS is shown in Fig. 2A. The bottom outline of the PTMS was designed to be a curve contour that is unparallel with the cell culture surface to obtain the PPFC alike flow field. Theoretically, the flow pattern of the viscous fluid within 2 unparallel surfaces should also obey the parabolic flow assumption. To obtain the relationship between WSS on the cell culture surface and the aspirate fluid volume flow rate *Q* on the outlet of the PTMS, we further performed the theoretical derivations of the relationship between WSS and *Q* based on the hydrodynamic principle (the continuity principle of the fluid). The fluid formulas at the radius *r_x_* of the bottom surface of the PTMS can be expressed as follows (where we assumed the mean velocity at the radius *r_x_* is ${\bar{v}}_{x}$, and the distance between the PTMS and the cell culture surface at the radius *r_x_* is *h_x_*, and the PTMS was placed vertically on top of the cell culture surface):

$$h_{x}{\bar{v}}_{x}=\int_{-\frac{h_{x}}{2}}^{\frac{h_{x}}{2}}\mathrm{Vdh}=\int_{-\frac{h_{x}}{2}}^{\frac{h_{x}}{2}}\left(-\frac{4V_{\max\_x}}{h_{x}^{2}}h^{2}+V_{\max\_x}\right)\mathrm{dh}$$ (1)

$${\bar{v}}_{x}=\frac{2}{3}V_{\max\_x}$$ (2)

$$Q=2\pi r_{x}h_{x}{\bar{v}}_{x}$$ (3)

$$Q=\frac{4}{3}\pi r_{x}h_{x}V_{\max\_x}$$ (4)

$$V_{\max\_x}=\frac{3}{4\pi r_{x}h_{x}}Q$$ (5)

As mentioned above, the WSS on the surface for a parabolic flow is equal to $4\mu \frac{V_{\max}}{h_{x}}$; therefore, WSS at the radius *r_x_* of the PTMS can be expressed as follows:

$$\mathrm{WSS}=\frac{3\mu }{\pi r_{x}h_{x}^{2}}Q$$ (6)

In this study, we aim to make the WSS remain constant along the radial direction above the cell culture surface when given a specific *Q*. Therefore, the value of $\frac{3\mu }{\pi r_{x}h_{x}^{2}}$ should be kept constant. Hence, we will have the following expression:

$$\frac{3\mu }{\pi r_{x}h_{x}^{2}}=\frac{3\mu }{\pi r_{0}h_{0}^{2}}$$ (7)

where the distance from the outer contour of the PTMS to the cell culture surface is called *h*_0_. Therefore, we can have the following expressions:

$$r_{x}h_{x}^{2}=r_{0}h_{0}^{2}$$ (8)

$$h_{x}=\sqrt{\frac{r_{0}}{r_{x}}h_{0}}$$ (9)

Therefore, the curve line $h_{x}=\sqrt{r_{0}/r_{x}}\cdot h_{0}$ was used to design the micro-structure of the head end of the PTMS.

### The fabrication process of PTMS

The 3D model of the PTMS is shown on the right of Fig. 2A, which was fabricated through precision machining using stainless steel material (SUS316) provided by Dongguan Yejia Precision Machinery Co., Ltd. Moreover, in order to minimize the effects of roughness on the final product, the surface of the PTMS was meticulously prepared, achieving a smooth finish with a surface roughness of less than 0.4 μm.

### Numerical scheme

CFD provides a reliable and effective approach to accurately analyze the hydrodynamics of complex structural systems. In this study, we simulated the flow field distribution of both the PTMS and the PPFC system using the CFD method. To solve the Navier–Stokes and continuity equations, we employed the finite volume method via the CFD software. Additionally, we utilized the SIMPLE algorithm to manage the pressure–velocity coupling and a second-order upwind scheme for the momentum spatial discretization in a coupled solver process.

### Adhered cells (colonies) selection by using the PTMS-FLOW

To pick up adhered cells (or colonies), we controlled the PTMS-FLOW acting time to 1 s using a PTMS and set the aspirate time of the pump accordingly. The fluid volume flow rate *Q* was controlled by the Tech Pump, which can produce a stable WSS (0 to 1,000 Pa) on the plate surface. The PTMS required an accurate *Z* distance (*h*_0_ = 50 μm) above the bottom of the culture plate to generate PTMS-FLOW for adhered cells (colonies) selection. The autofocus device was used to calculate the *Z* distance. The *X* and *Y* coordinates were generated by the computer vision system to guide the robotic arm selection operation. The adhered cells included NPCs, iPSCs, and UCs.

### Ethics statement

The mouse model was maintained and cared for in our Experimental Animal Centre’s facility in accordance with the Guangzhou Institutes of Biomedicine and Health Institutional Animal Care Using the Committee protocols. All experiments were performed in accordance with the guidelines set by the Human Subject Research Ethics Committee at the Guangzhou Institutes of Biomedicine and Health and the Chinese Academy of Sciences. The committee approved all experiments. Formal informed consent was obtained from all the subjects.

### Statistical analysis and reproducibility

Two-tailed paired or unpaired Student’s *t*-test was performed for analyses using GraphPad Prism software. The significance levels are *P < 0.05, **P < 0.01, ***P < 0.001, and ****P < 0.0001.

## Data Availability

All data needed to evaluate the findings are present in the paper and/or the Supplementary Materials. Their expression matrix information was deposited in National Genomics Data Center (NGDC) of China National Center for Bioinformation with the number PRJCA013662 and PRJCA014685. The code and the case examples for image analysis and for generating the location information of colonies were located at the GitHub link (https://github.com/bpkcl630/iPScCloneIdentification).
