# Supplementary material for: Selecting Monoclonal Cell Lineages from Somatic Reprogramming Using Robotic-Based Spatial-Restricting Structured Flow
Source: Research (Wash D C). 2024 Mar 7;7:0338. doi: 10.34133/research.0338 (PMC10923610; doi:10.34133/research.0338)
Supplement: Supplementary 1 — Figs. S1 to S10 Tables S1 to S3 Movies S1 to S3 [file research.0338.f1.zip › Supporting Information.pdf]

Supporting Information

Selecting Monoclonal Cell Lineages from Somatic Reprogramming using Robotic-based Spatial-restricting Structured Flow

Xueping Chen[1†], Ke Fan[1†], Jun Lu[1,6†], Sheng Zhang[1†], Jianhua Dong[1†], Jisheng Qin[1†], Weihua Fan[1], Yan Wang[1], Yiyuan Zhang[1], Huo Peng[1], Zhizhong Zhang[1], Zhiyong Sun[1], Chunlai Yu[7], Yucui Xiong[1], Yan Song[1], Qingqing Ye[1], Shiwen Mai[1], Yuanhua Wang[1], Qizheng Wang[1], Fengxiang Zhang[1], Xiaohui Wen[1], Tiancheng Zhou[1], Li Han[3], Mian Long[2], Guangjin Pan[1], Julian F. Burke[5], Xiao Zhang[1,4*]

Supplementary figures

Fig. S1 to S10 for multiple supplementary figures

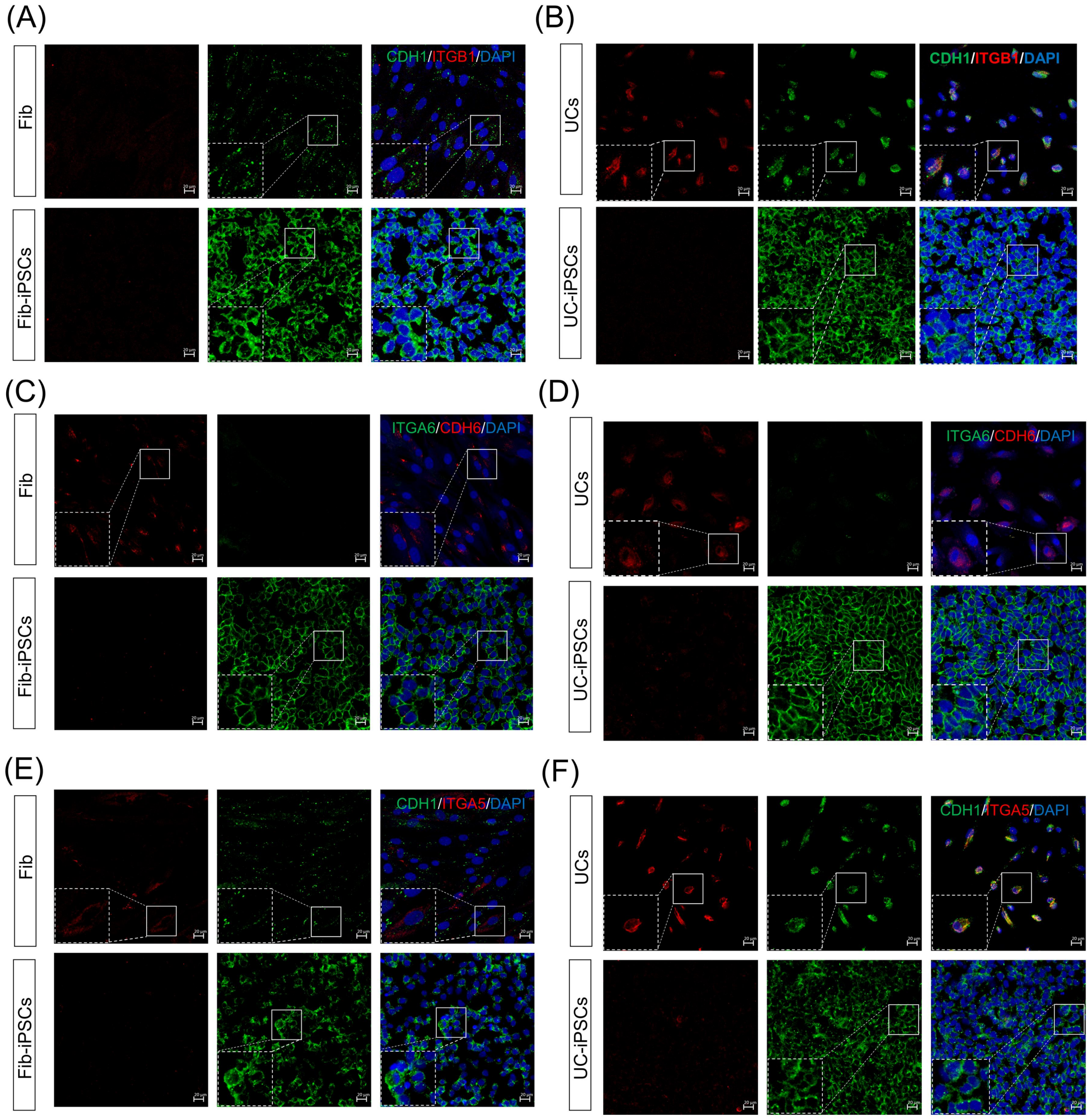

Fig. S1: The expression of cadherin and integrin gene panel were altered during the process of re-programming. (A-F) Immunofluorescence staining analysis between somatic cells (UCs: urinal cells, Fib: fibroblast) and their derived iPSCs using integrins (ITGB1, ITGA5, and ITG6) and cadherins (CDH1, CDH6), scale bar is 20 μm.

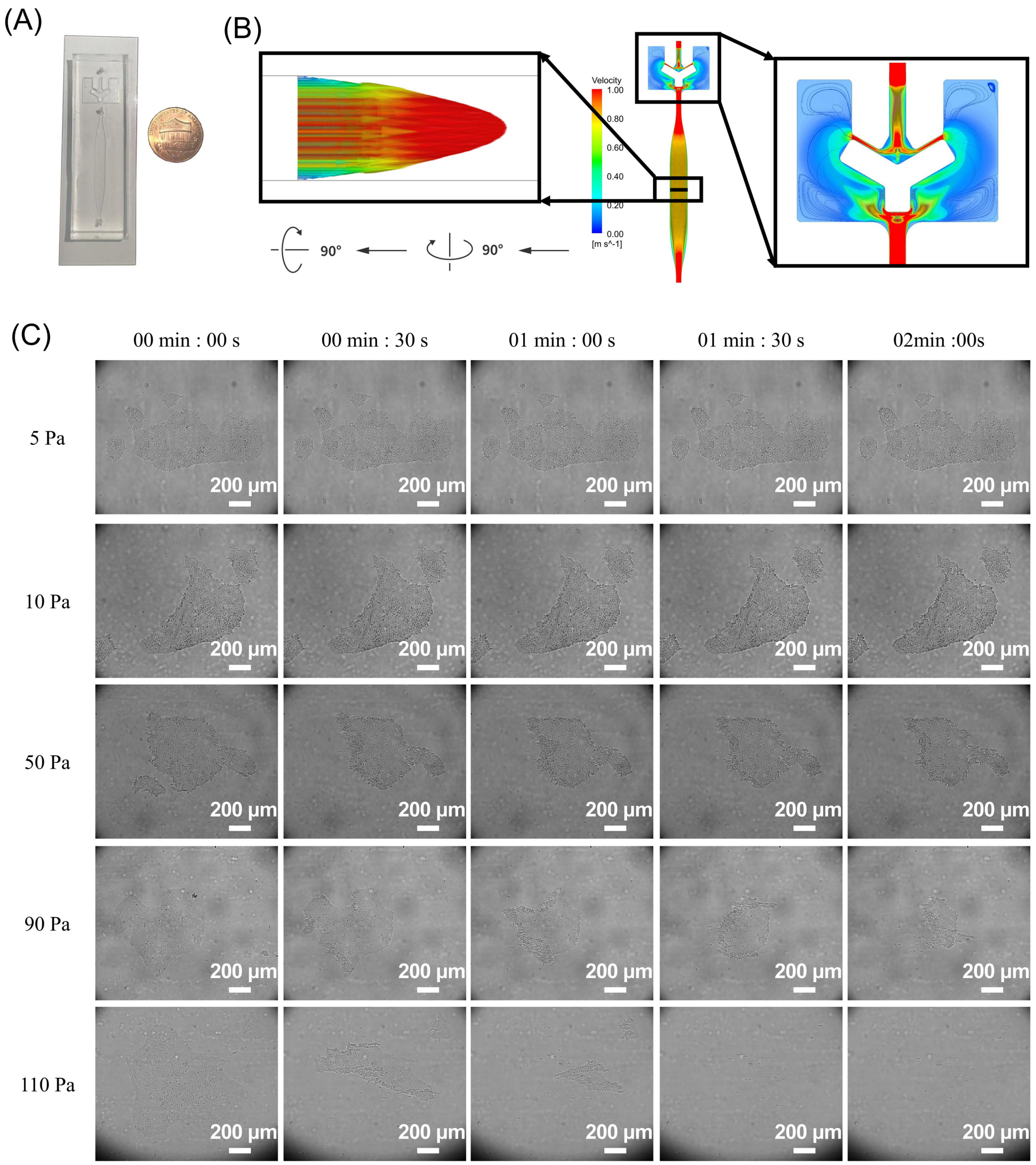

Fig. S2: The in-house developed Parallel-Plate Flow Chamber (PPFC) experiments. (A) Picture of the PPFC device. (B) The CFD simulation results of the PPFC structure. (C) The results of the fluid shear stress-induced iPSCs colony dissociation. The area of the colony dissociation was a positive correlation with the magnitude of fluid wall shear stress (WSS); in contrast, the time required for colony complete dissociation was reduced with the increase of WSS.

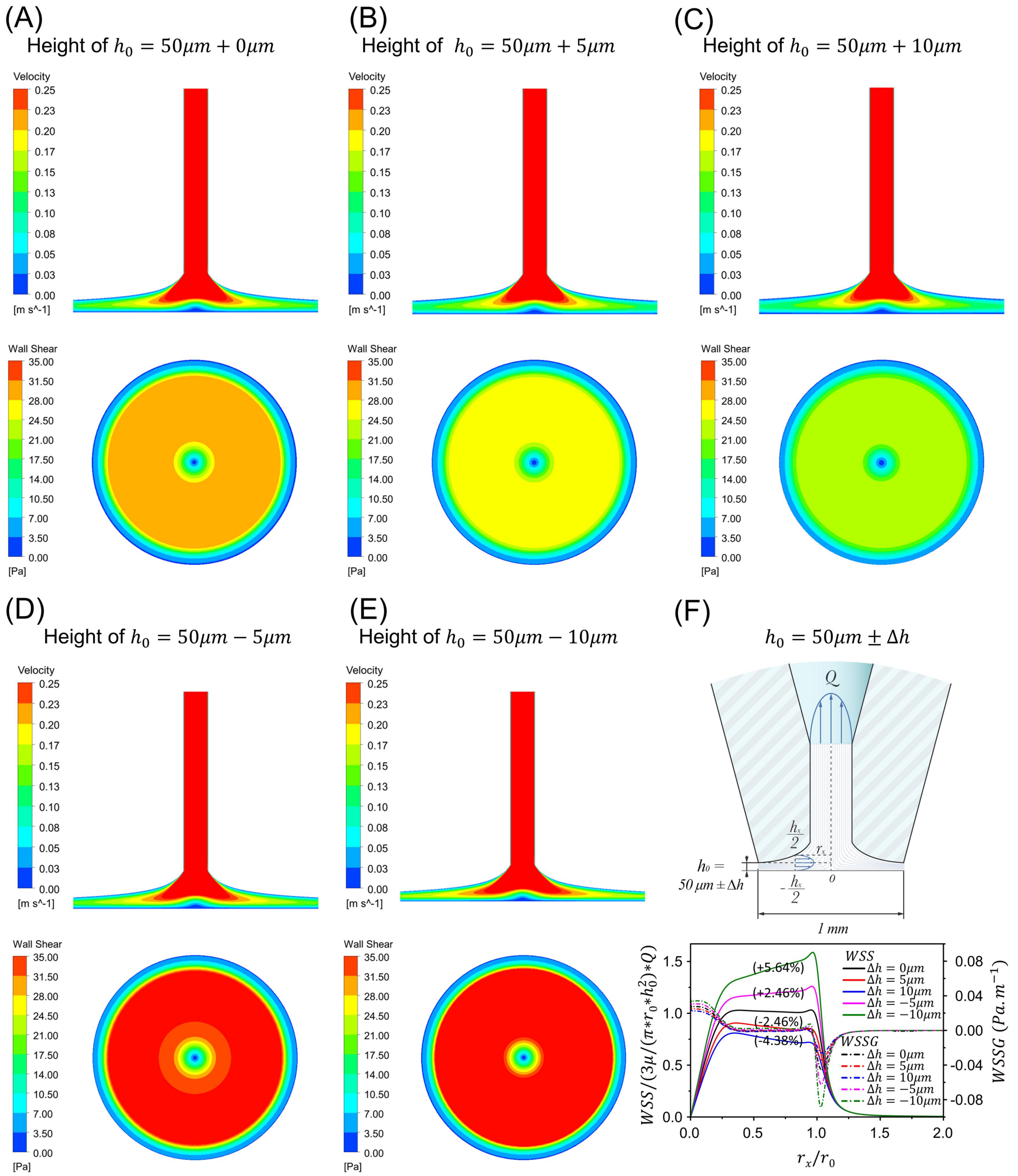

Fig. S3: The absolute value change of WSS can be retained within 3% when h_0 restrained within ±5 μm. (A-F) The comparison of CFD simulation result on the PTMS with the Z distance variation of +5 μm, +10 μm, -5 μm, -10 μm, respectively. The absolute value change of WSS was found to be within 3% when the parameter h_0 was restrained within ±5 μm.

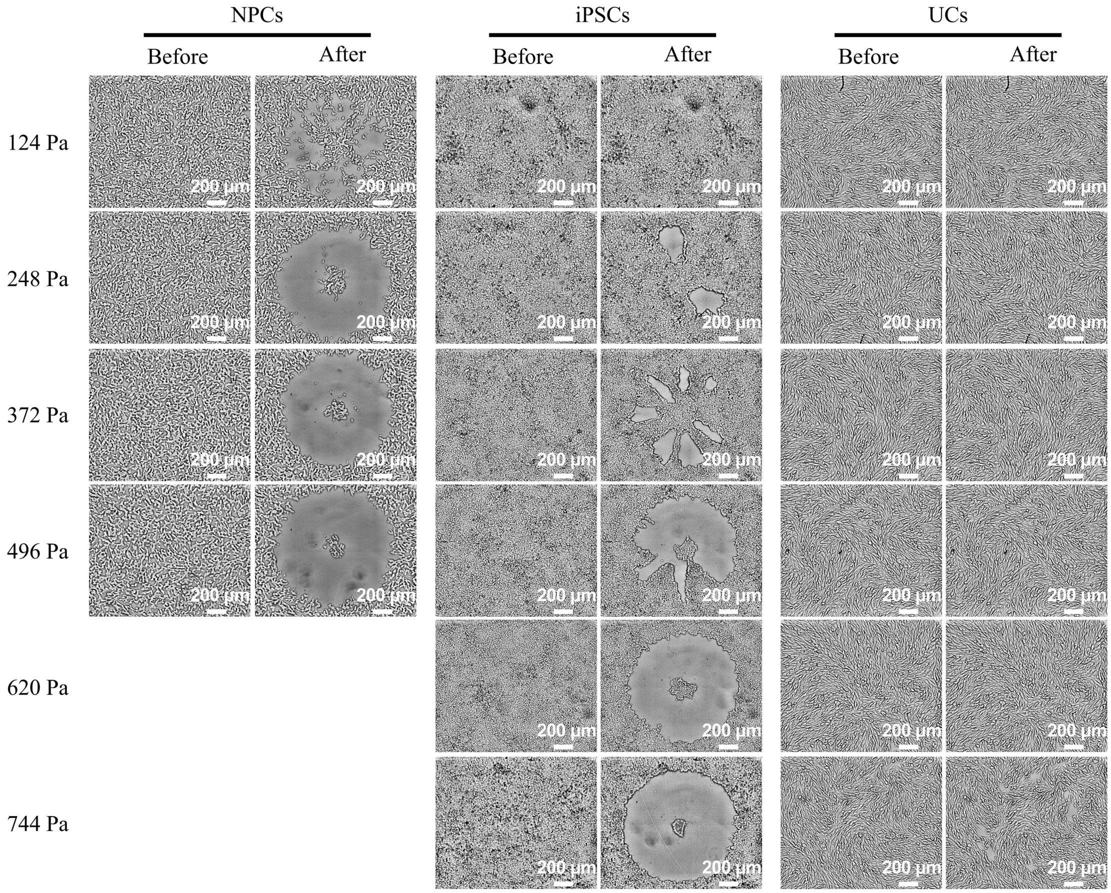

Fig. S4: The area of the colony dissociation for three representative cell lineages (NPCs, iPSCs, and UCs) with various PTMS-FLOW-based shear stress generated by the PTMS. Under the same fluid shear stress, NPCs showed the lowest adhesion strength, followed by iPSCs and UCs, respectively.

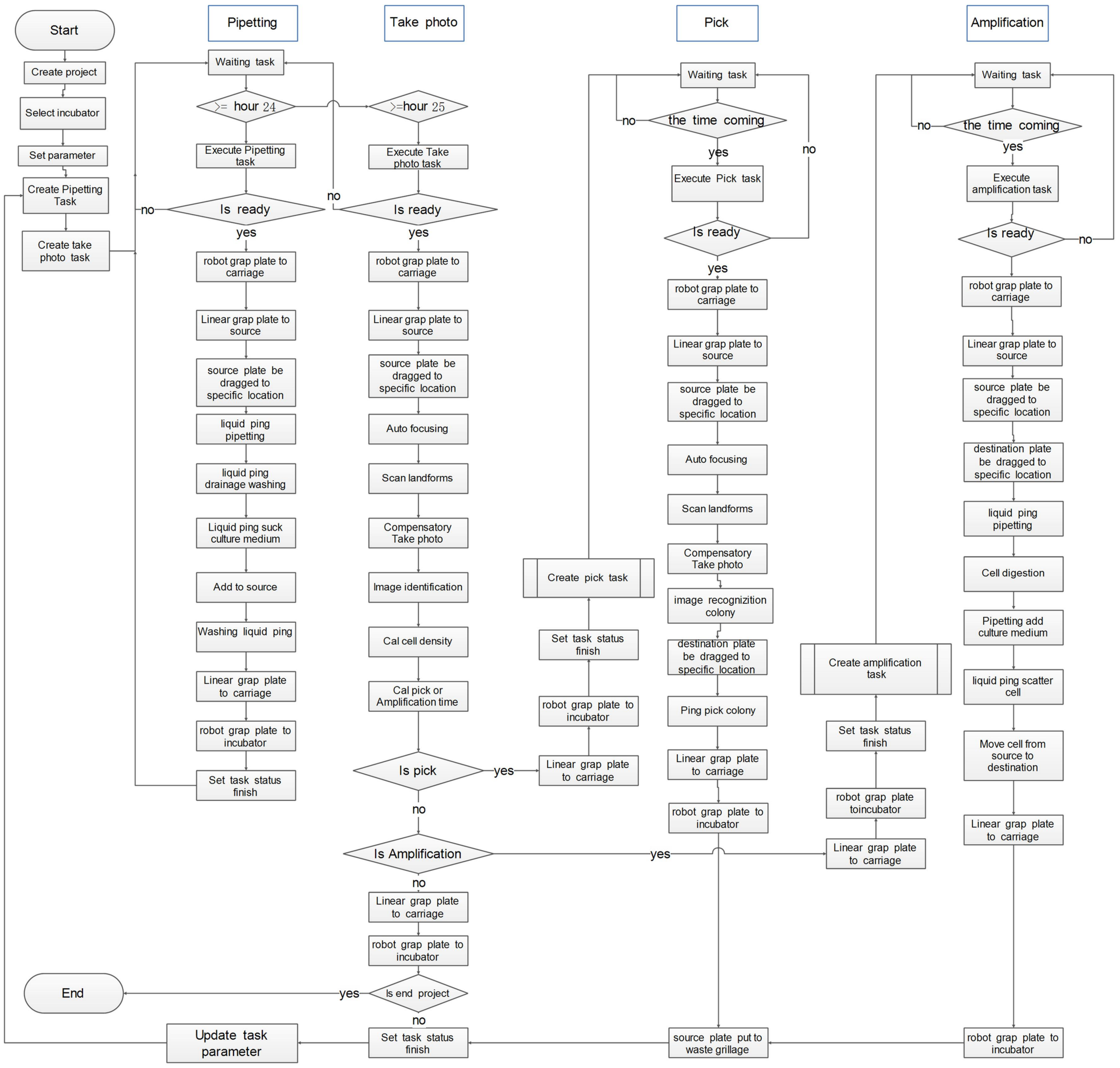

Fig. S5: Detailed workflow of iPSCs colony selection and culture system.

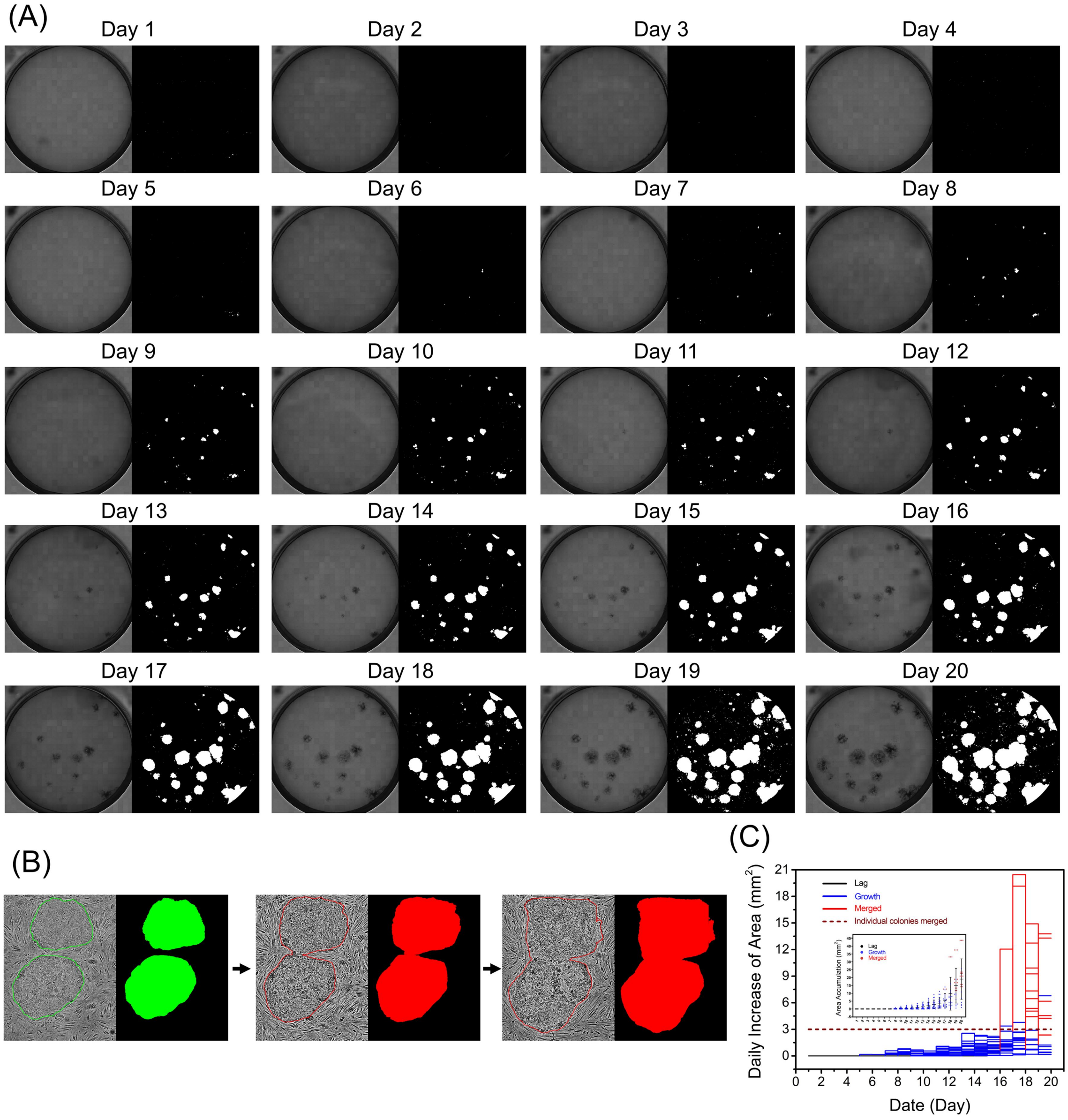

Fig. S6: The "read-on-ski" imaging technology for colonies detection. (A) Daily images were recorded to track the colonies during reprogramming (B) The example images depict the merging of two adjacent colonies. (C) The daily increasing in colony area (DIA) revealed consistent changes over time. Ï$IA = A\,rea_{Day(N)} - A\,rea_{Day(N-1)}$. Various colors signify different growth phases; black, blue, and red represent the Lag phase, Growth phase, and Merged phase, respectively. The dashed line serves as a reference value of ÏAI for distinguishing between the Growth phase and the Merged phase. The inset shows the combined growth of individually selected colonies.

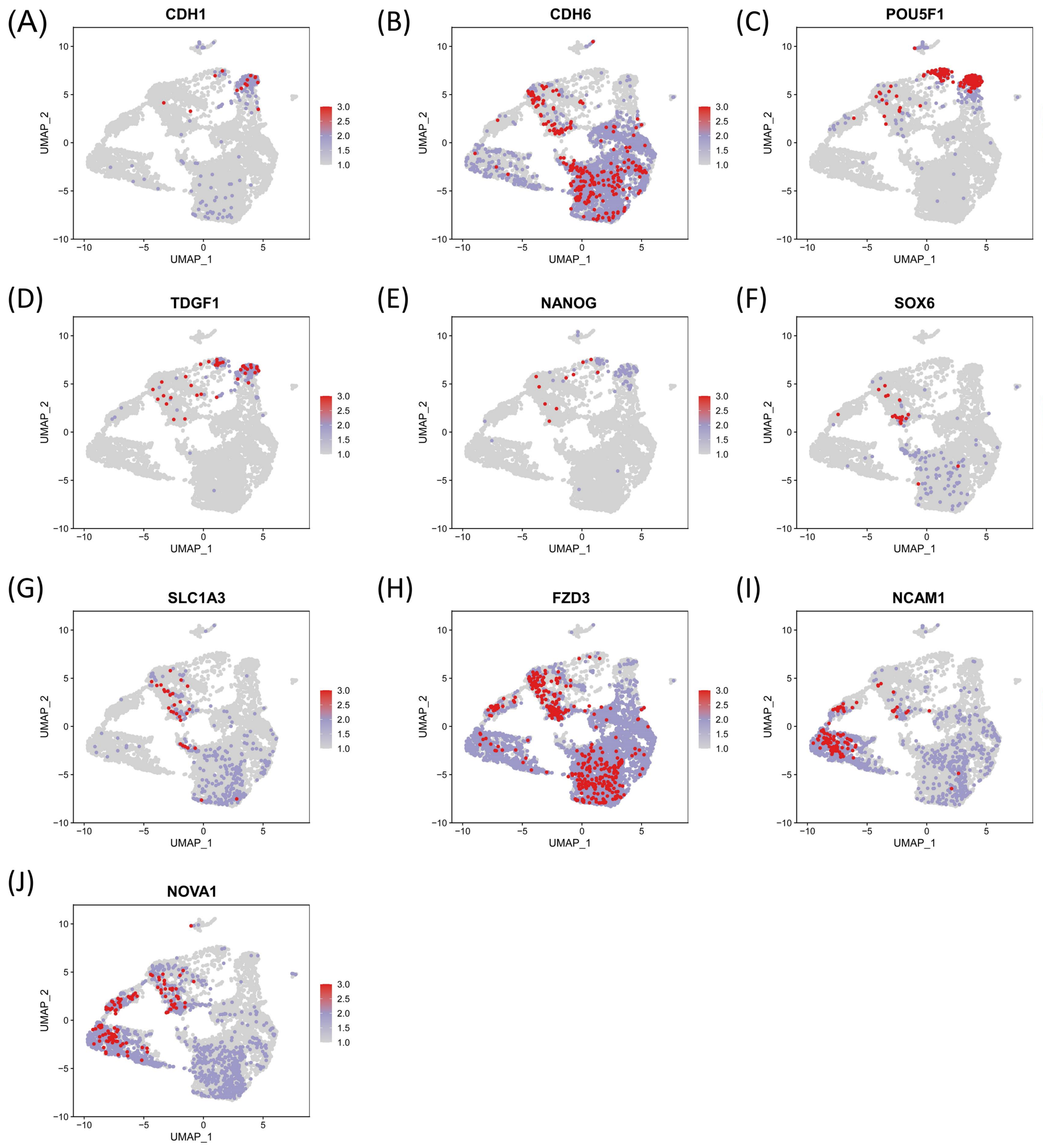

Fig. S7: UMAP plot exhibiting expression pattern of markers of the selected colonies through manual selection. Cadherin gene CDH1 and CDH6 are separately expressed in iPSCs and NPCs (A&B).

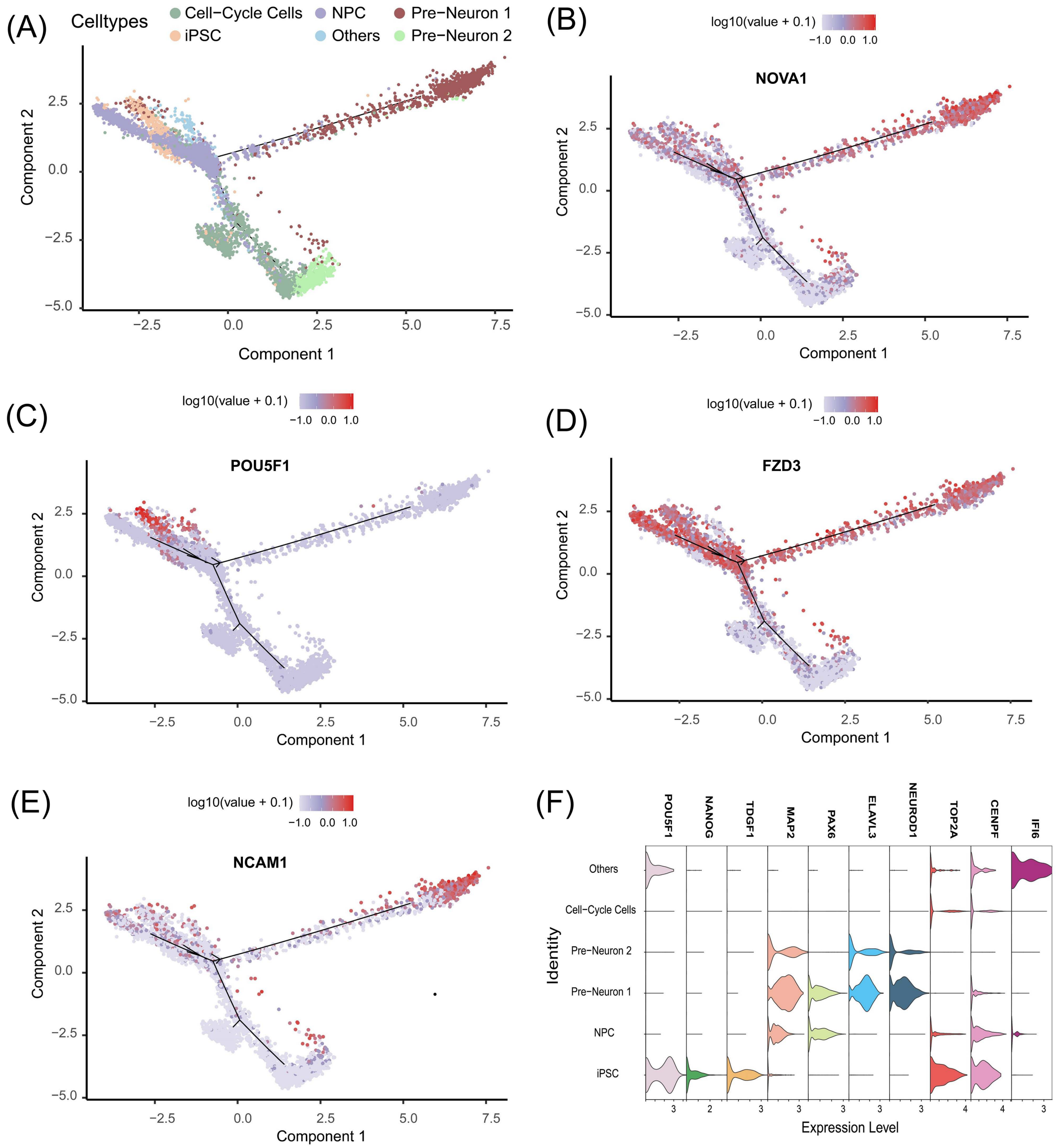

Fig. S8: The scRNA-seq pseudotime trajectory analysis during somatic cell reprogramming. (A) The pseudotime trajectory of cell types in reprogrammed colonies based on manual selection. (B)-(E) The pseudotime trajectory plots the expression of genes related to cell fate. Expression of NOVA1, POU5F1, FZD3 and NCAM1 along the lineage development. (F) The marker gene expression of different cell types.

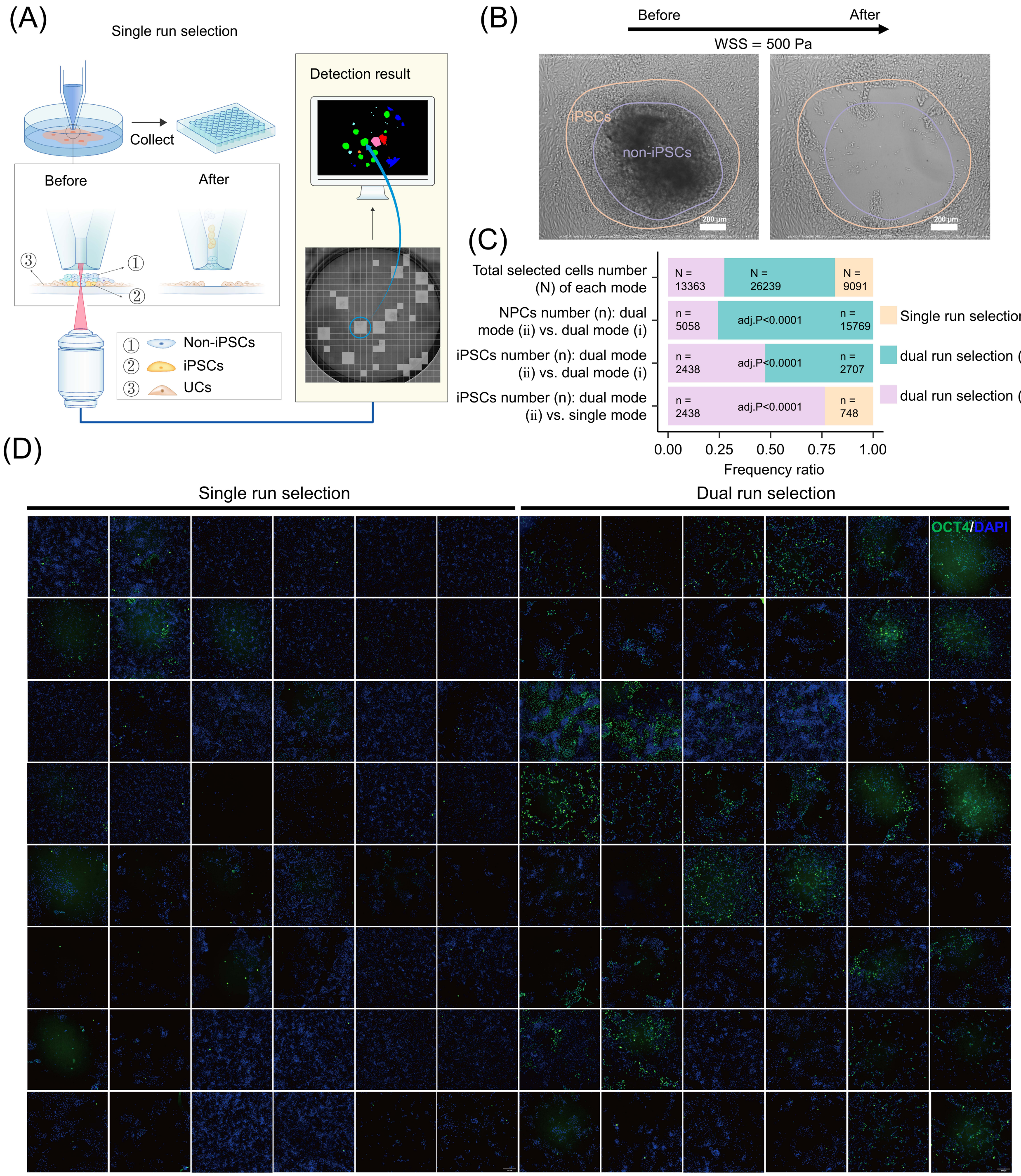

Fig. S9: Comparing the robustness of different selection modes for obtaining colonies. (A) Schematic representation of the single selection mode. (B) The bright-field image of selected colonies using the single selection mode. Scale bar is 200 μm. (C) The comparison of the cell numbers of NPCs and iPSCs obtained through three different selection modes. (i) stands for WSS=250 Pa, and (ii) for WSS=500 Pa. (D) The performance validation of the selection strategy in the batch mode. All selected colonies were subjected to OCT4 staining, with the results being compared between the single run selection mode (Left) and the dual run selection mode (Right), Scale bar is 200 μm.

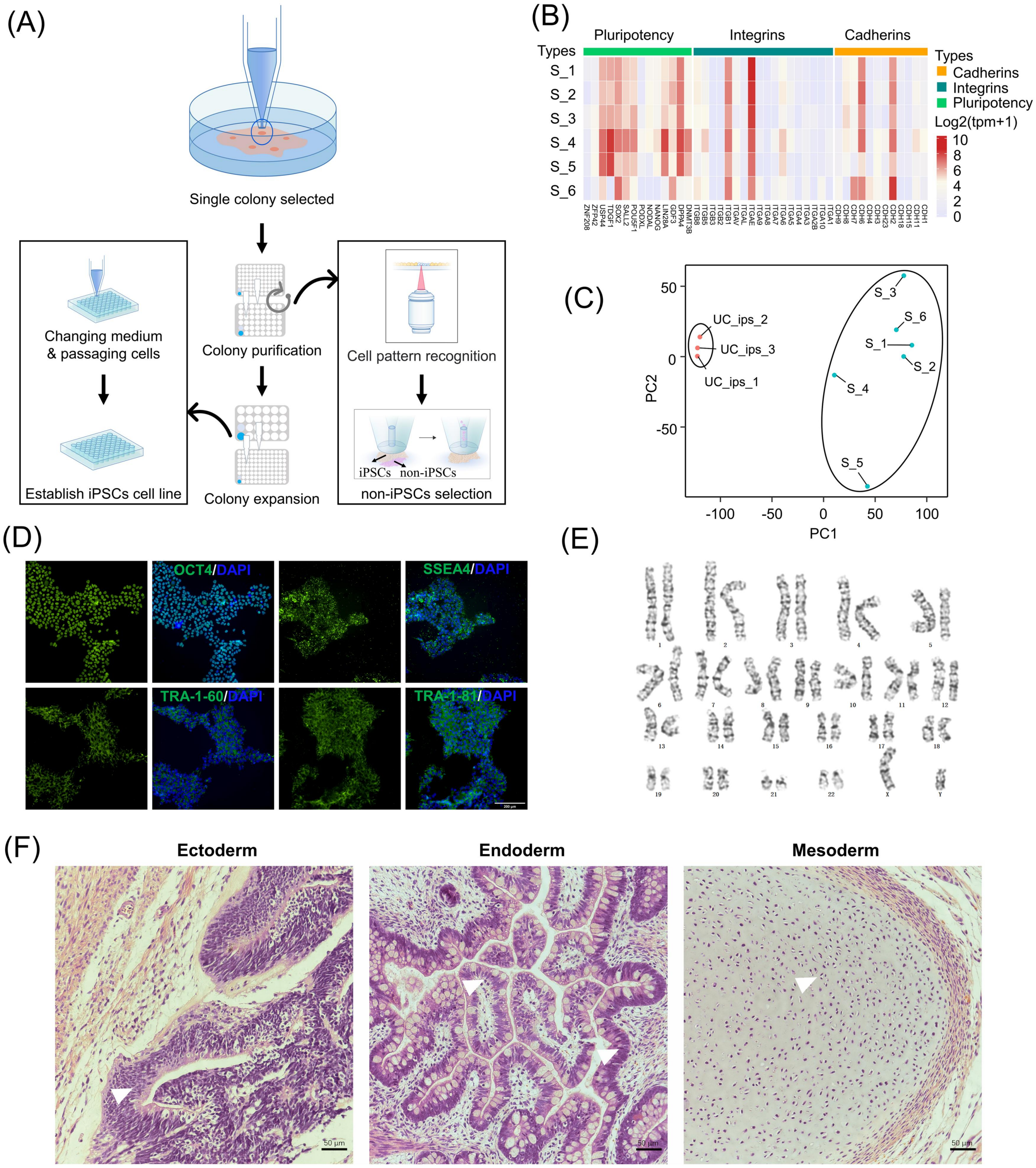

Fig. S10: The selected iPSCs were biologically characterised using standard stem cell approaches. (A) Schematic representation of the negative selection approach used for the colony purification. (B) Analysis of the cell types of the negatively selected cells during the purification process. Gene list is same as Fig. 1B. (C) Principal component analysis (PCA) was conducted to compare the negatively selected cells with the UC-iPSCs during the purification process. (D) OCT4, TRA-1-60, and SSEA4 staining of the iPSCs after automation facilitated dual-selection, purification, and expansion. Scale bar is 200 μm. (E) The identification of the karyotypes of the iPSCs after automation facilitated dual-selection, purification, and expansion. (F) The pluripotency confirmation of the iPSCs selected by the robotic system, after injection into SCID mice. Three white arrows point to the corresponding embryonic germ layers. Scale bar is 50 μm.

Supplementary tables

Table S1 to S3 for multiple for the supplementary tables

Table S1: Frequency ratio of specific cell type among four selecting modes.

Selection Modes	NPCs	iPSCs	Pre-Neuron 1	Pre-Neuron 2	Cell-Cycle Cells	Others
Manual	0.565	0.048	0.125	0.054	0.195	0.013
Single run	0.657	0.083	0.077	0.011	0.152	0.020
Dual run (i)	0.601	0.103	0.012	0.006	0.275	0.002
Dual run (ii)	0.379	0.182	0.016	0.002	0.406	0.015

Table S2. Comparison of the superiority of our robotic system to other technologies and developments (from existing commercial products).

Commercial Products / Technical Points	FACS/FLOW	ClonePix	CellCelector	Berlkley Lights	μSHEAR Device	In this study
Automated iPSCs generation (post transfection)	✗	✗	✗	✗	✗	✓
Label-free	✗	✗	✓	✓	✓	✓
Enzyme free	✗	✓	✗	✓	✓	✓
Non-invasive	✗	✗	✗	✗	✓	✓
High through-put	✓	✓	✗	✓	✗	✓
Action in selection speed	✓	✓	✗	✗	✗	✓
Data traceability	✗	✓	✗	✗	✗	✓
Selection with monoclonality as an individual clone	✗	✓	✓	✓	✗	✓
Imaging assurance of monoclonality	✗	✓	✓	✓	✓	✓
Adaptive in the selection variation of FSS	✗	✗	✗	✗	✗	✓
Multiple selections using different FSS at the same clone	✗	✗	✗	✗	✗	✓
Apply under the ordinary culturing condition	✗	✓	✓	✗	✗	✓

Notes: ✓ means this Technical Point can be implemented. ✗ means this Technical Point cannot be implemented. FSS: fluid shear stress.

Table S3: The list of the antibodies used in the paper.

Antibody	Host	Item No.	Brand	Ratio
OCT4	Ms	sc5279	Santa Cruz	1/200
OCT4	Rb	2750	CST	1/200
FZD3	Rb	ab21703	Abcam	1/200
NOVA1	Rb	ab97368	Abcam	1/200
ITGB1	Rb	4706	CST	1/400
ITGA5	Rb	10569	Proteintech	1/250
ITGA6	Rat	sc19622	Santa Cruz	1/1000
CDH1	Ms	610181	BD	1/200
CDH6	Rb	48111	CST	1/800

Supplementary Videos

Video S1 to S4 for multiple supplementary Videos

Video S1: The CFD simulation results showed that the designed flow field effectively entraps dissociated cells within the collection area.

Video S2: The observed dissociation behaviour of the colony exhibited a continuous interconnected pattern with cells adhering to one another while detaching from the culture surface.

Video S3: The trans-illumination based "read-on-ski" imaging technology. The green and red colors respectively represent the clones before and after merging.

Video S4: The robotic system working process.
